# Impact of a novel exponential weighted 4DCT reconstruction algorithm

**DOI:** 10.1002/acm2.12423

**Published:** 2018-09-11

**Authors:** Eric D. Morris, Joshua P. Kim, Paul Klahr, Carri K. Glide‐Hurst

**Affiliations:** ^1^ Department of Radiation Oncology, Henry Ford Cancer Institute Detroit MI USA; ^2^ Department of Radiation Oncology, Karmanos Cancer Center Karmanos Cancer Center, Wayne State University School of Medicine Detroit MI USA; ^3^ Computed Tomography and Advanced Molecular Imaging Business Unit, Philips Healthcare Cleveland Ohio USA

**Keywords:** 4DCT, artifacts, reconstruction

## Abstract

**Purpose:**

This work characterizes a novel exponential 4DCT reconstruction algorithm (EXPO), in phantom and patient, to determine its impact on image quality as compared to the standard cosine‐squared weighted 4DCT reconstruction.

**Methods:**

A motion platform translated objects in the superior–inferior (S‐I) direction at varied breathing rates (8–20 bpm) and couch pitches (0.06–0.1) to evaluate interplay between parameters. Ten‐phase 4DCTs were acquired and data were reconstructed with cosine squared and EXPO weighting. To quantify the magnitude of image blur, objects were translated in the anterior–posterior (A‐P) and S‐I directions for full‐width half maximum (FWHM) analysis between both 4DCT algorithms and a static case. 4DCT sinogram data for 10 patients were retrospectively reconstructed using both weighting factors. Image subtractions elucidated intensity and boundary differences. Subjective image quality grading (presence of image artifacts, noise, spatial resolution (i.e., lung/liver boundary sharpness), and overall image quality) was conducted yielding 200 evaluations.

**Results:**

After taking static object size into account, the FWHM of EXPO reconstructions in the A‐P direction was 3.3 ± 1.7 mm (range: 0–4.9) as compared to cosine squared 9.8 ± 4.0 mm (range: 2.6–14.4). The FWHM of objects translated in the S‐I direction reconstructed with EXPO agreed better with the static FWHM than the cosine‐squared reconstructions. Slower breathing periods, faster couch pitches, and intermediate 4DCT phases had the largest reductions of blurring with EXPO. 18 of 60 comparisons of artifacts were improved with EXPO reconstruction, whereas no appreciable changes were observed in image quality scores. In 18 of 20 cases, EXPO provided sharper images although the reduced projections also increased baseline noise.

**Conclusion:**

Exponential weighted 4DCT offers potential for reducing image blur (i.e., improving image sharpness) in 4DCT with a tendency to reduce artifacts. Future work will involve evaluating the impact on treatment planning including delineation ability and dose calculation.

## 1 INTRODUCTION

The advent of four‐dimensional computed tomography (4DCT) has enabled a more accurate characterization of the internal target volume (ITV)^1^, ^2^, ^3^ of moving tumors while reducing motion artifacts.^4^, ^5^ 4DCT offers the ability to characterize tumor motion and therefore reconstruct its trajectory over a patient's breathing phase.^6^ However, current 4DCT reconstruction approaches have some limitations. Geometric inaccuracies may arise from the interplay between tumor and couch motion. This could cause instances during data acquisition where the gantry is rotating slower than a specific anatomical change, where the temporal resolution is no longer sufficient to represent patient anatomy.^7^ It has been shown that as the temporal window is widened to collect more projection data, volumes will become more distorted, along with an increasing amount of image artifact.^8^ Furthermore, including too much projection data has been shown to blur moving anatomy,^9^ thus leading to poor definition at object boundaries.^7^ In this study, we evaluate the impact of applying, in phantom and patient, an exponential weighting factor to the current standard of care (i.e., cosine‐squared weighting factor) in order to generate an exponential 4DCT reconstruction algorithm (“EXPO”). Early work presented by Shen et al. suggested that using EXPO yielded improved volume estimation and reduced motion artifact in 4DCT.^10^ Our work builds upon these preliminary results by presenting a detailed theoretical basis for the algorithm, evaluates its performance in controlled phantom experiments with a large variety of acquisition parameters, and then performs a quantitative and qualitative comparison between conventional and EXPO 4DCT reconstructions for a patient cohort, with the overarching goal of evaluating the potential of using EXPO reconstruction to sharpen the boundaries of moving targets for radiation therapy.

## 2 MATERIALS AND METHODS

### 2.A Exponential reconstruction

One of the main challenges in 4DCT is the ability of the scanner to capture an adequate amount of data during the entire respiratory cycle to accurately represent patient anatomy in each phase. When using a helical scan, it is imperative that the pitch factor be set low enough so that the entire scan volume, including the voxels at the edge of the field of view (FOV), are illuminated throughout the respiratory cycle.^11^

**Figure 1:**
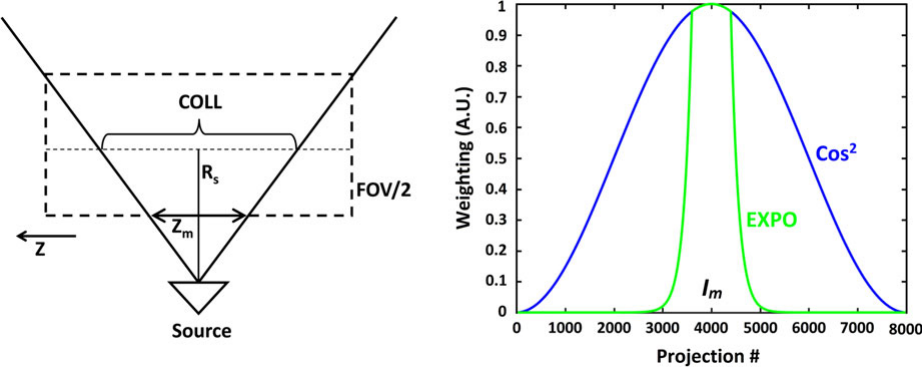
Left: Schematic of the CT geometry illustrating the relationship between the source to CT isocenter distance (R_S_) traversing along the z axis, with respect to the collimator width (COLL) and field of view (FOV) as described in the text. Right: Curves of cosine squared and EXPO weighting functions revealing how weighting algorithm varies around mid‐phase point *I*_*m*_.

Figure 1 (left) illustrates the distance (*Z*_*m*_) along the axis of couch motion (*z*‐axis) that is visible to the collimators at the edge of the FOV, which can be written as:

(1) $$Z_{m}=\frac{\left(R_{S}-\text{FOV}/2\right)\times \text{COLL}}{R_{S}}$$

where R_S_ is the source to CT isocenter distance and COLL is the collimator width. Couch velocity can be expressed as $V=\frac{\left(\text{PF}\times \text{COLL}\right)}{\text{RT}}$, where RT is the rotation time and PF is the pitch factor. Therefore, the transit time (TT) at which the voxel traversing the *z*‐axis at the edge of the FOV remains within the FOV is determined by $\text{TT}=Z_{m}/V$.

For the entire FOV to be illuminated throughout the breathing cycle, the TT for the voxel traversing along the line *Z*_*m*_ must be greater than the breathing period (BP). This then specifies a condition for the BP:

(2) $$\text{BP}\leq \frac{\left(R_{s}-\text{FOV}/2\right)}{R_{s}}\times \frac{\text{RT}}{\text{PF}}$$

In terms of PF, this condition becomes

(3) $$\text{PF}\leq \left(1-\text{FOV}/2R_{s}\right)\times \frac{\text{RT}}{\text{BP}}$$

when the PF is too large, this condition is violated, and the reconstruction software widens the temporal gate to ensure that the minimum criteria for the number of sinogram projections is met to accurately reconstruct the pixels at the edge of the FOV. The increase in the time window (*T*_*w*_) for reconstruction can be derived using:

(4) $$T_{w}=\text{BP}-\left(\frac{\text{RT}}{\text{PF}}\left(1-\frac{\text{FOV}}{2R_{s}}\right)\right)$$

where *T*_*w*_ is temporal increase in seconds. A minimum of π (half‐rotation) projections are required to accurately reconstruct a CT image. When eq. (3) is satisfied, the reconstruction system can utilize π projections centered at each phase point and temporal resolution will be optimized. When the gating window is broadened because the pitch is not low enough, some pixels will be reconstructed from more than π projections. For these pixels, this represents redundant data, which may degrade temporal resolution as described in detail by Manzke et al.^12^ To minimize this impact on the temporal window, a cosine‐squared weighting factor is commonly used and, when applied to the *i*th projection, is given by^12^:

(5) $$W_{t}\left(i\right)=\cos^{2}\left(\frac{\left(i-I_{m}\right)}{I_{t}}\right)$$

where $I_{t}$ = total projections and $I_{m}$ = mid‐phase point. However, using cosine‐squared weighting employs a gradual downward slope that causes projections far from the reconstruction point (in a temporal sense) to contribute significantly to image reconstruction. Thus, we propose to multiply the cosine‐squared weighting factor with an exponential weighting function:

(6) $$\begin{array}{c} \text{Exponential term}={\text{e}}^{-\left(\frac{\left(\text{abs}\left(i-I_{h}\right)\right)}{I_{t}}\right)}E_{f} \end{array}$$

where $I_{h}$ = number of projections and $E_{f}$ controls the curve's steepness. A constant value of $E_{f}=2.0$ was previously selected as the clinical default based on motion platform experiments performed during algorithm development at the manufacturer factory. This value was empirically determined to be the point at which motion artifacts were no longer visible. This parameter is not customizable and will not be altered in clinical scenarios after vendor optimization was performed. The proposed final weighting function, or EXPO, can now be given as:

(7) $$\text{EXPO}=\cos^{2}\left(\frac{\pi \left(i-I_{m}\right)}{I_{t}}\right)*{\text{e}}^{-\left(\frac{\text{abs}\left(i-I_{h}\right)}{I_{t}}\right)E_{f}}$$

This sharper slope (Fig. 1, right) is expected to improve the magnitude of image blurring by minimizing the weighted contribution of outside projections. Thus, it is our hypothesis that by implementing the exponential weighting factor into the 4DCT reconstruction, image sharpening and edge enhancement will be achieved relative to the conventional cosine‐squared reconstruction technique. Additionally, it is expected that the attenuation of redundant data will cause an increase in image noise.

### 2.B Phantom research methods

**Figure 2:**
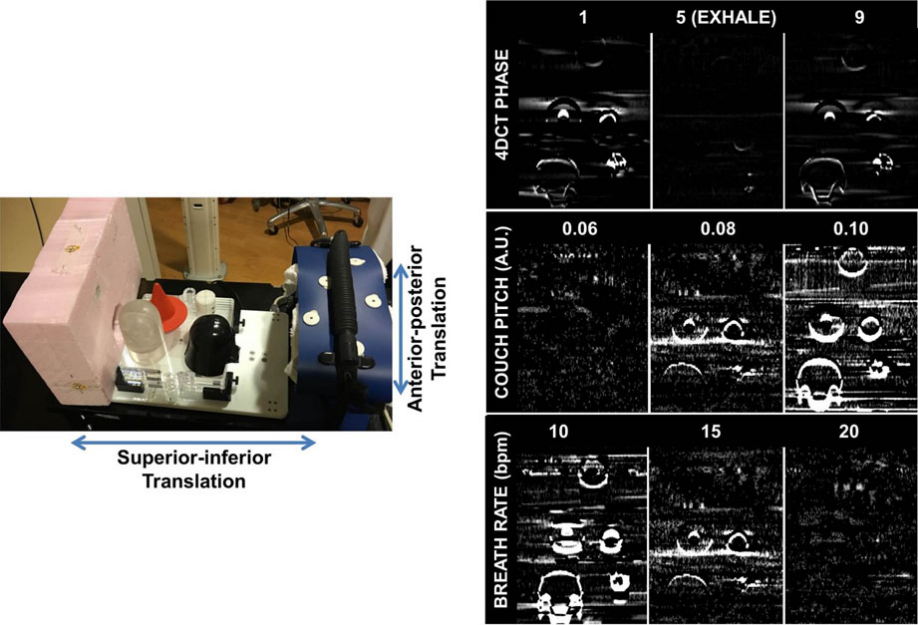
Left: Phantom experimental setup. The pneumatic belt is placed around the anterior to posterior excursion surrogate, whereas the objects of varied densities, shapes, and sizes were placed on the platform moving in the superior–inferior direction using a sinusoidal waveform. Right: Difference maps of phantom experiment between cosine squared and exponential (EXPO) reconstruction algorithm for several acquisition parameters. Difference maps were calculated using cosine squared less EXPO.

Phantom experiments were carried out using a commercially available programmable motion phantom (ExacTrac Gating Phantom, Version 1.0, BrainLAB AG, Germany). Several objects of varying size and contrast were embedded in a lung‐mimicking Styrofoam slab and placed on the motion platform to be translated in the superior–inferior (S‐I) direction, as shown in Fig. 2 (left). The bellows pneumatic belt was placed around a platform on the phantom that moves synchronously in the anterior–posterior (A‐P) direction to derive the breathing waveform used for 4DCT sorting. To simulate respiratory motion, ten‐phase 4DCT scans using phase‐based sorting were acquired with the following acquisition parameters: sinusoidal waveform at couch pitches of 0.06, 0.08, and 0.10 (unitless), and breathing rates of 10, 12, 15, and 20 bpm. CT images were acquired with the phantom held at a stationary location at the end‐inhale (EI), end‐exhale (EE), and a mid‐point position (i.e., static positions) to serve as ground truth data. For all phantom and patient experiments described in this work, a large‐bore 16‐slice CT scanner was used with a 3‐mm slice thickness and 120 kVp (Brilliance™ CT Big Bore v3.6; Philips Health Care, Cleveland, OH).

As a means to estimate the difference in magnitude of image blur between standard and experimental reconstructions, a methodology was followed in a manner similar to McCollough et al.^13^ Here, the magnitude of image blur was estimated via the measurement of the full‐width half maximum (FWHM) of a small moving object. To fully characterize EXPO, the FWHM were calculated for objects translated in both the S‐I (sinusoidal pattern for parameters described above) and the A‐P (sawtooth pattern) directions. Sawtooth waveform data were acquired at breathing rates of 8, 10, and 12 bpm, with couch pitches of 0.06, 0.08, and 0.10. The magnitude of image blur was calculated by taking the FWHM less the known static object size for the reconstructions at each 4DCT phase.

All raw 4DCT data were reconstructed using both the standard cosine squared and experimental (exponential) weighting factors. Image subtraction images for each phase were then generated to elucidate intensity and boundary differences. Maximum intensity projection images (MIPs), minimum intensity projections (minIPs) and average CTs (AVG‐CTs) were generated using in‐house MATLAB (version R2014b) software previously described.^14^

### 2.C Research method for human subject studies

A retrospective analysis was performed by reconstructing raw 4DCT datasets for 10 lung, breast, and abdomen patients using both standard cosine squared and exponential weighting factors as part of an Institutional Review Board approved study. A variety of breathing rates and tumor locations were evaluated with couch pitches varying from 0.06 to 0.141. Difference maps were generated via image subtraction of the individual phases and derivative images (i.e., MIPs, minIPs, and AVG‐CTs) to investigate differences in intensity and edge effects. These derivative images were generated using an in‐house script (MATLAB, version 2014b). Regions of interest near visible tumors were evaluated for local changes.

Three Medical Physicists with experience in 4DCT formed a consensus group and performed subjective image quality grading for the 10‐patient cohort, which represented various respiratory waveforms, breathing rates, couch pitches, anatomical regions, and patient sizes. All 4DCT images were oriented in the coronal plane, and the standard cosine squared and exponential weighted reconstructions were displayed simultaneously (ImageJ, version 1.43u, available at: http://rsb.info.nih.gov/ij). Patient data were anonymized and uploaded in a random order to maintain a blinded study. Patients were evaluated for the presence of liver dome artifacts and overall image noise, using the following 4‐point image grading scale^15^, ^16^: (a) negligible impact, (b) minor impact without relevance for clinical evaluation, (c) major impact causing clinical evaluation to be difficult, and (d) significant impact rendering image not suitable for clinical use. To assess liver dome artifacts, the EI, EE, mid‐EE (30%), mid EI (70%), as well as MIP and minIP images were graded. Overall image noise was evaluated using the EI phase and a region of interest (ROI) comparison was conducted for six anatomical regions for the patient case with the worst noise score. Spatial resolution (i.e., sharpness of liver/lung interface) was scored with a similar 4‐point grading scale: (a) negligible blurring, (b) slight blurring without relevance for clinical use, (c) moderate blurring with possible relevance for clinical use, and (d) completely blurred boundary. EI and minIP images were scored for spatial resolution. Finally, AVG‐CTs, which are commonly used for dose calculation and treatment planning in 4DCT cases,^3^ were graded for overall image quality. Over all metrics and phases, 200 evaluations were conducted.

## 3 RESULTS

### 3.A Phantom results

Figure 2 (right) summarizes the major findings of the phantom experiments. In general, reconstructions performed with a slow breathing rate (8–10 bpm) yielded the largest differences, particularly near object boundaries. Similarly, as the couch pitch increased, the differences between the resulting exponential and cosine‐squared reconstruction images also showed a tendency to increase. As shown in Fig. 2 (right, top), differences were also observed between the individual mid‐inhale and mid‐exhale phase reconstructions. No apparent differences occurred at the end‐exhale phase where the waveform and motion tend to be moving with the least amount of velocity. At transitional phases, however, differences between reconstruction algorithms were observed with similar behavior existing between phases with similar speeds on the waveform (for example, phase 1 and phase 9).

**Figure 3:**
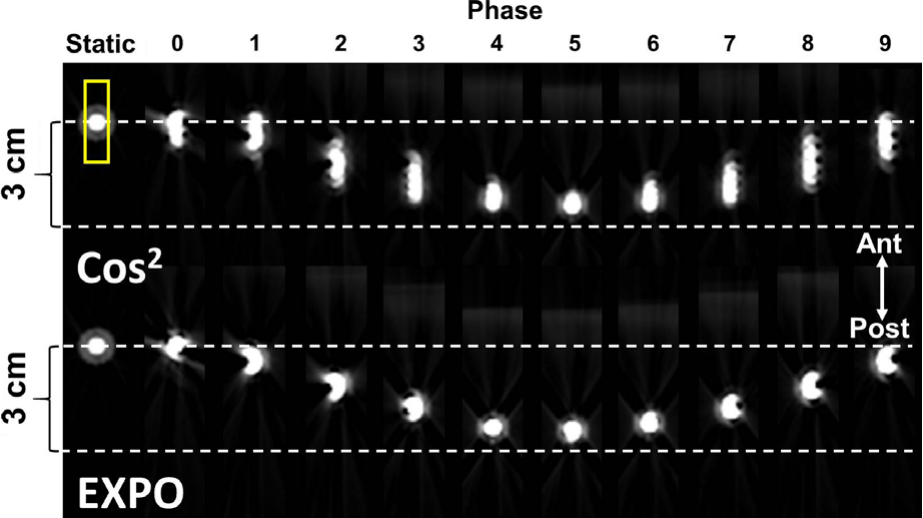
*Anterior–posterior* moving object reconstruction results for a small high contrast object placed on a programmable motion phantom image quality evaluation and full‐width half maximum calculations using the region of interest shown in the top left. Top Row: Standard of care cosine‐squared reconstructions, Bottom Row: Experimental exponential reconstructions. Data were acquired with a sawtooth waveform (12 bpm, pitch = 0.08, excursion 3 cm). The static object size is shown in each row for comparison. (Phase 0 = end‐inhale, Phase 5 = end‐exhale).

**Figure 4:**
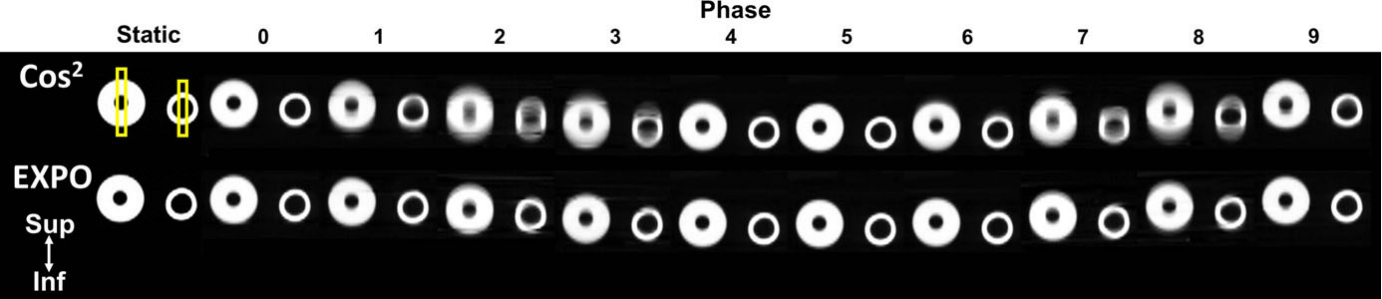
*Superior–inferior* moving object reconstruction results for two small high contrast objects for image quality and full‐width half maximum calculations using the region of interest shown in the top left. Top Row: Standard of care cosine‐squared reconstructions, Bottom Row: Experimental exponential reconstruction algorithm results. Images were acquired using a sinusoidal waveform (12 bpm, pitch = 0.08, excursion = 3 cm). The static object size is shown in each for comparison purposes. (Phase 0 = end‐inhale, Phase 5 = end‐exhale).

Figures 3 and 4 highlight the greatest detected improvement by using EXPO. The magnitude of image blur improved (i.e., FWHM was reduced) when using EXPO across all 4DCT phases. In all phantom studies, EXPO reconstructions agreed more closely with the object's static size (shown on left‐most column of Figs. 3 and 4). In assessing the object moving in the A‐P direction (Fig. 3), after subtracting the static object size (7.3 mm), the remaining FWHM of the exponential weighting was 3.3 ± 1.7 mm (range: 0–4.9), as compared to the FWHM of cosine squared [9.8 ± 4.0 mm (range: 2.6–14.4)]. For both EXPO and cosine‐squared reconstructions, the end‐exhale phase (i.e., phase 5) had the closest FWHM to the static size (7.4 mm and 9.9 mm, respectively).

Similar results were observed when assessing objects translated in the S‐I direction as highlighted in Fig. 4. For the larger object shown on the left side, after subtracting the static object size (54.4 mm), the remaining FWHM of EXPO was 0.5 ± 0.3 (range: 0.1–1.0), as compared to the FWHM of cosine squared [1.4 ± 1.2 (range: 0.3–3.3)]. Furthermore, for the smaller object shown on the right, after subtracting the static object size (36.7 mm), the remaining FWHM for EXPO was 1.1 ± 0.7 (range: 0.1–2.1), as compared to the FWHM of cosine squared [4.6 ± 5.0 (range: 0.1–13.9)]. For both the S‐I and A‐P directions, the largest differences were observed for the intermediate phases (i.e., 2–3, 7–9), where the excursion and image blur were most marked.

### 3.B Results for human subject studies

**Figure 5:**
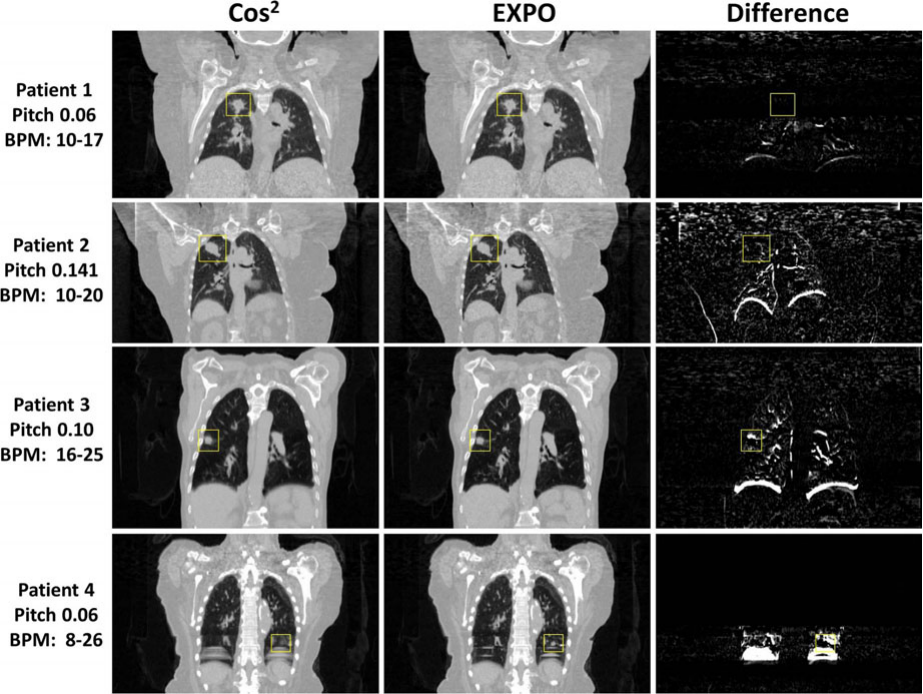
Retrospective reconstructions using the standard of care (cosine squared) and experimental (exponential, or EXPO) reconstruction algorithms for four different lung cancer patient 4DCTs for the end‐inhale breathing phase (0%). Difference maps were calculated using cosine‐squared less EXPO and boxes indicate the location of the tumor regions treated. The largest improvement was observed for Patient 4, where EXPO significantly decreased the use of incorrect projections in the phase reconstruction and increased lesion conspicuity substantially.

The retrospective patient analysis yielded similar results as the phantom experiments. The coronal data shown in Fig. 5 best illustrates the image differences between the reconstruction algorithms for a subset of cases and their associated parameters showing minor differences, average differences, image sharpening around tumor, and increased tumor visualization, respectively. Note the significant reduction in blurring observed near the lung/diaphragm and lung/liver interfaces for all patient cases when EXPO reconstruction was implemented. In rows 3 and 4, there is an increased sharpness and visibility of the tumor (outlined by the ROI). For Patient 4, using the EXPO reconstruction showed a significant reduction in image artifact in the diaphragm region. Of note, the tumor (outlined by the box) was not detectable in the cosine‐squared reconstruction but became clearly visible with the use of EXPO. For most cases, the baseline noise also slightly increased due to the use of fewer sinograms in the reconstruction. Nevertheless, clear local intensity differences can be detected near mobile anatomy such as the tumor region, at times increasing lesion conspicuity, or at the liver/lung interface.

**Figure 6:**
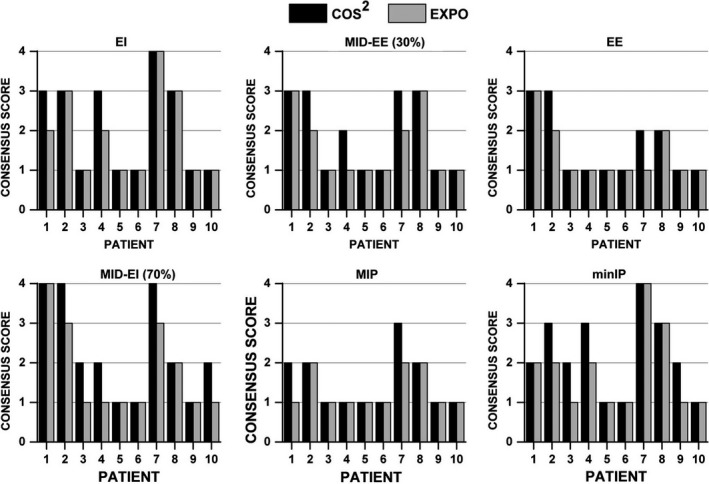
Qualitative consensus grading (3 observers) for the presence of liver dome artifacts using the following image grading scale: 1. negligible impact, 2. minor impact without relevance for clinical evaluation, 3. major impact causing clinical evaluation to be difficult, and 4. significant impact rendering image not suitable for clinical use. Abbreviations: EI = end‐inhale, EE = end‐exhale, MIP = maximum intensity projection, minIP = minimum intensity projection.

The qualitative image grading revealed several noteworthy results. For all 10 patients reviewed, there was no change in overall image quality scores for the AVG‐CT between reconstruction methods. Of 20 comparisons of image sharpness for the EI and minIP datasets, two patient cases had equivalent scores, whereas for the other 18, EXPO was selected as providing a sharper image. For three of these cases, cosine‐squared reconstructions yielded an image that had “Moderate blurring with possible relevance for clinical use” whereas no EXPO reconstructions scored worse than a 2. However, when the EI images were evaluated for image noise, all cosine‐squared reconstructed images were given a grade of 1, whereas EXPO reconstructions were deemed noisier with a grade of 2 for half of the patients studied. The grainier images tended to be derived from patients with larger pitches (0.08–0.141). Results for the image artifact evaluation are best summarized by Fig. 6.

Overall, of 60 total comparisons of image artifacts, EXPO reconstructions reduced artifacts for 18 cases relative to cosine‐squared reconstructions. One patient case (Patient 7), with results shown in Fig. 6, revealed that EXPO reconstructions were improved in 4/6 of the artifact parameters studied. For 3 of 10 patients, qualitative consensus grading revealed equivalent scores between cosine squared and EXPO for all images evaluated for the presence of artifacts. Typical causes for this were that the patients had faster breathing rates (20–27 bpm), the 4DCT acquisition was of very poor quality (cosine squared and EXPO were both given scores of 4), or the 4DCTs were of very high quality (i.e., Patients 5 and 6 were given a score of 1 in every case and had regular/periodic waveforms). Figure 6 also highlights that image artifact scores were equivalent between images reconstructed with cosine squared and EXPO for 70% (42 of 60) of the datasets. As expected, patients with high‐quality, cosine‐squared 4DCT reconstructions did not yield artifact improvement with the use of EXPO, with scores of 1 (negligible impact) observed in 28/60 matched pairs across phases. In rare cases with severe artifacts with a consensus score of 4 (significant impact rendering image not suitable for clinical use), using EXPO did not reduce the artifacts. This is likely because the window of image projections used could not be narrowed enough for significant artifact improvement while still maintaining an adequate signal to noise ratio in the image. Nevertheless, Patient 4 did show a reduction in major artifacts that improved tumor visibility when EXPO was employed (Fig. 5). Of the 4DCT phases evaluated, EE contained the most scores of 1. This is likely due to the elongated and stable position at the end‐exhalation breathing phase. When taking the patient's breathing waveform into account, regardless of pitch or breathing rate, the patients that showed improvements in the most individual phases when using EXPO tended to have several irregularities present in their respiratory waveform. Overall, the impact of exponential reconstructions on derivative images such as the AVG‐CT, minIP, and MIP datasets revealed only slight differences in image artifact with comparable image quality.

**Figure 7:**
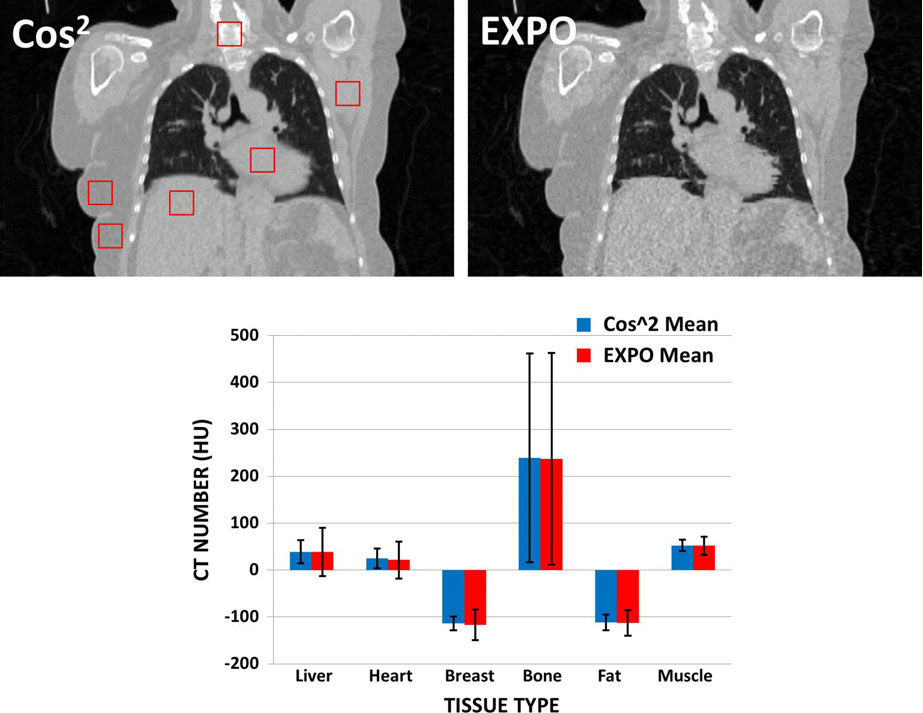
Region of interest analysis conducted for the patient yielding the largest discrepancy in image noise content during image quality review. Top row: Cosine‐squared weighted reconstruction (left) revealing examined ROIs and EXPO weighted reconstruction (right) scored 3 for noise in image quality review. Bottom: Mean measured CT number in ROIs across varying tissues for cosine squared and EXPO weighted reconstruction algorithms.

Figure 7 highlights ROI results for a patient who yielded an increased noise score when EXPO reconstruction was used. Overall, negligible differences in the mean CT number were observed for each ROI (~2.0 HU, range: 0.9–3.6 HU). In general, the standard deviation increased among all tissues types except for bone. The largest increase in standard deviation was observed for the liver (~27 HU). This can be observed in both the measurements and in the underlying image quality shown in Fig. 7. This case can be considered the worst‐case scenario due to large body habitus secondary to the patient's body mass index and treatment position (one arm up for breast treatment).

## 4 DISCUSSION

This study sought to introduce a novel exponential weighting factor for 4DCT reconstruction and evaluate its performance against the current clinically implemented cosine‐squared reconstruction algorithm. The phantom evaluation revealed that the impact of the exponential weighting factor increased during transitional 4DCT phases, at increased pitches, and at decreased breathing rates. In assessing the magnitude of image blur for the phantom, the exponential weighting algorithm yielded more accurate estimations of object size across all 4DCT phases evaluated when compared to cosine‐squared reconstructions. The image quality evaluation of patient data revealed that overall, EXPO reconstructions showed a tendency to reduce visible 4DCT artifacts, increase sharpness at image boundaries, and in some cases, yield noisier images.

Our phantom work was consistent with preliminary data presented by Shen et al., who performed 4D phantom experiments at two pitch settings that revealed significant improvements when using EXPO with a higher pitch (0.1) and less of an improvement at a pitch of 0.06 for a breathing rate of 12 BPM.^10^ Although a similar result was seen in our phantom data, these results did not translate to patient cases where more individualized results were observed, suggesting an interplay of the different parameters including couch pitch, breathing regularity, and breathing rate. In our study, one result of the phantom experiment that translated to patient cases was that the differences between EXPO and cosine‐squared reconstructions were less apparent with rapid breathers such as patients 5, 6, and 8 shown in Fig. 6, with mean breathing rates of 20–27 bpm. For both the phantom and patient evaluations, when the breathing rate increased, the impact of EXPO was less prominent. With higher breathing rates, the reconstruction algorithm utilizes the temporal window constrained to its minimum of π projections and thus, a weighting scheme is not employed. This suggests that patients with the slowest breathing rates will be most impacted by EXPO, which is also confirmed by the phantom reconstructions where the slowest breathing rates (8–10 bpm) yielded the largest differences, particularly near object boundaries. When reconstructed with EXPO, Patient 4, with very irregular breathing ranging from 8 to 26 BPM over the course of the 4DCT acquisition, had a marked reduction in artifacts and the tumor became more visible as highlighted by Fig. 5. Generally speaking, patients with irregularities in their respiratory waveforms had a tendency to show the most improvement in individual phases when using EXPO. In addition, Fig. 6 reveals that EXPO reconstructions improved liver dome artifacts in half of the cohort at the mid‐EI (70%) respiratory phase, which agrees with the phantom results that the impact of EXPO is increased at transitional phases (Figs. 3 and 4). This suggests that EXPO has potential to impact patients with irregular breathing patterns and in clinics that utilize all 4DCT phases in their treatment planning workflows.

To our knowledge, few 4DCT reconstruction algorithms that are similar to EXPO are available for comparison. Half of the reconstructions were scored as slightly noisier in the qualitative image quality grading when EXPO was used due to the decreased number of projections used in the reconstructions. However, when quantified, the largest difference in standard deviation was ~27 HU for a liver, whereas the differences in mean HU for each tissue type was generally negligible (<2.0 HU). To put this into clinical context, ~20 HU difference for soft tissue yields ~2% difference in electron density, and a 4–10% electron density difference results in a ~2% difference in dose calculation assuming 6 MV photons are used.^17^ Nevertheless, appreciable differences in image sharpness were observed in 85% of the images reviewed with a substantial reduction in 4DCT artifact through the inclusion of fewer projections. Important next steps of this work are to include physician delineation analysis to determine if the tradeoff in image sharpness and reduction of 4DCT artifacts with the slightly increased image noise render the images suitable for contouring, although this will largely depend on disease site. However, delineations have been shown to be prone to a great deal of inter‐ and intraobserver variability,^17^ and thus it may be difficult to decouple the source of contouring differences. Thus, the work conducted here was an important first step toward clinical implementation.

Our work revealed that 4DCT phases with little to no motion, such as end‐exhalation, are least affected by the reconstruction algorithm. This suggests that clinics using the end‐exhalation phase for respiratory‐gated deliveries may not benefit from EXPO reconstructions.^18^, ^19^ In a similar manner, derivative images such as the MIP and AVG‐CT were not as sensitive to EXPO, likely due to the redundancy of data among the 10 different breathing phases offsetting any impact on individual 4DCT phases. This work included only phase‐based 4DCT sorting. It is possible that amplitude‐based sorting may produce different results which can be explored in future work.

Overall, our analysis of both phantom and patient data showed that the EXPO reconstruction algorithm improved image sharpness while decreasing image artifacts. A potential clinical implementation may be to reconstruct raw data using both reconstruction techniques and building hybrid patient models to take advantage of the increased sharpness provided in high motion areas, whereas preserving signal in the noise‐starved conditions typically present in the abdominal and liver regions.

## 5 CONCLUSION

4DCT reconstructions generated with EXPO offered reductions in image blur and motion artifacts. This, in turn, leads to more edge enhancement at anatomical boundaries, particularly in highly mobile anatomical sites. While a slight increase in baseline noise was observed, understanding the potential implications on delineation ability and treatment planning are important next steps of this work.

## CONFLICT OF INTEREST

Henry Ford Health System holds research agreements with Philips Healthcare. Paul Klahr is a clinical scientist employed at Philips Healthcare.
